# Non-equilibrium quantum phase transition via entanglement decoherence dynamics

**DOI:** 10.1038/srep34804

**Published:** 2016-10-07

**Authors:** Yu-Chen Lin, Pei-Yun Yang, Wei-Min Zhang

**Affiliations:** 1 Department of Physics and Centre for Quantum Information Science, National Cheng Kung University, Tainan 70101, Taiwan

## Abstract

We investigate the decoherence dynamics of continuous variable entanglement as the system-environment coupling strength varies from the weak-coupling to the strong-coupling regimes. Due to the existence of localized modes in the strong-coupling regime, the system cannot approach equilibrium with its environment, which induces a nonequilibrium quantum phase transition. We analytically solve the entanglement decoherence dynamics for an arbitrary spectral density. The nonequilibrium quantum phase transition is demonstrated as the system-environment coupling strength varies for all the Ohmic-type spectral densities. The 3-D entanglement quantum phase diagram is obtained.

In quantum many-body systems, quantum phase transitions (QPTs) may occur at zero temperature as a parameter varies in the Hamiltonian of the system, induced purely by quantum fluctuations^1^. QPTs have been explored via entanglement^2^,^3^,^4^,^5^,^6^, because entanglement is regarded as a key resource to detect QPTs, owing to the fact that the entanglement can exist without any classical correlations^7^. In previous investigations, QPTs are usually investigated in terms of entanglement for the many-body systems via the von Neumann entropy by dividing the system into various bipartites. In these investigations, significant changes of the entanglement as a parameter varies in the Hamiltonian of the system provide a deeper understanding about QPTs.

In order to experimentally explore QPTs, it is crucial how to manipulate the basic parameters in the Hamiltonian of the system, such as hopping energies and interaction coupling strengthes, etc. through the external devices. Thus, these systems manifesting QPTs become naturally open systems^8^,^9^,^10^. In this article, we attempt to explore nonequilibrium QPTs by studying the entanglement decoherence dynamics in a prototype open quantum system of two entangled modes interacting with a general non-Markovian environment. We find that entanglement decoherence dynamics, induced by the interaction between the system and its environment, manifests a significant change as the system-environment coupling strength varies from the weak to the strong coupling regimes. Nonequilibrium quantum phase transition occurs due to the competition between quantum dissipation dynamics and localization. Thermal fluctuations drive further the entanglement decoherence dynamics into a quantum critical regime and then into the thermal disordered regime. This could open a new venue for the experimental investigations of QPTs through the real-time entanglement decoherence dynamics.

## Results

### Real-time exact solution of entangled squeezed states

We consider a system with two entangled continuous variables, such as two entangled cavity fields, interacting to a common thermal environment, its dynamics is described by the Fano-Anderson Hamiltonian^11^,^12^,
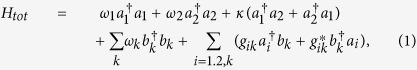
where *a*_*i*_ and 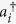
(i = 1, 2) are the annihilation and creation operators of the two continuous variables with frequency *ω*_*i*_, and *κ* is a real coupling constant between the two continuous variables, which can be tuned, for example, through a beam splitter on the two single-mode fields^13^. The environment Hamiltonian consists of an infinite number of bosonic modes, where *b*_*k*_ and 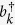 are the annihilation and creation operators of the mode *k* with frequency *ω*_*k*_. The interaction between the system and the environment is given by the last term in Eq. (1), and the parameter *g*_*ik*_ is the coupling amplitude between the continuous variable mode *i* and the environment mode *k*. The complexity of the problem is embedded in the spectral density, see Eq. (5) later, which characterizes the complicated energy structure of the environment plus the system-environment interaction.

In order to investigate the entanglement decoherence dynamics of the two continuous variables under the influence of a complicated environment, we take a decoupled initial state between the system and the environment^14^: *ρ*_*tot*_(0) = *ρ*_*s*_(0) ⊗ *ρ*_*E*_(0). The initial state of the system is *ρ*_*s*_(0) = |*ψ*_*s*_(0)〉〈*ψ*_*s*_(0)|, where |*ψ*_*s*_(0)〉 is an entangled state between the two continuous variables, and the environment is initially in thermal equilibrium 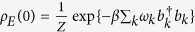 at the initial inverse temperature *β* = 1/*k*_*B*_*T*. After the initial time, the system and the environment both dynamically evolve into non-equilibrium states. To be specific, let *ρ*_*s*_(0) be a two-mode squeezed state^15^,
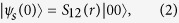
where 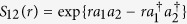 is a two-mode squeezed operator, and *r* is the real squeezing parameter. This state has been experimentally realized in many different systems, first given by Heidmann *et al*.^16^, and has also been applied to quantum teleportation^17^. When the squeezing parameter becomes very large, the above state would approach to the ideal Einstein-Podolsky-Rosen (EPR) state^18^.

Without loss of generality, we may consider the two continuous variables as two identical fields and interact homogeneously with the environment, namely, *ω*_1_ = *ω*_2_ = *ω*_0_ and *g*_*ik*_ = *g*_*k*_. Then we can introduce the center-of-mass and the relative motional variables, respectively, given by 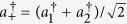, and 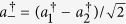, with the corresponding frequencies *ω*_±_ = *ω*_0_ ± *κ*. It is easy to check that only the center-of-mass variable *a*_+_ is coupled with the environment, and the relative-motional variable decouples from the environment which forms a decoherence-free subspace^19^. If the two continuous variables are not identically coupled to the environment, no such decoherence-free subspace exists, and the decoherence dynamics of the relative motion behaves similarly as that of the center-of-mass motion. In terms of the center-of-mass motion and the relative motion of the two continuous variables, we can rewrite the initial state (2) as a direct product of two entangled states, |*ψ*(0)〉 = |*ψ*_+_(0)〉 ⊗ |*ψ*_−_(0)〉, where
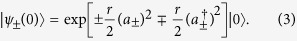


Because the relative motion *a*_−_ is decoupled from the environment, the entanglement between the two continuous variables in |*ψ*_−_(0)〉 is decoherence-free. Therefore, the entanglement decoherence only happens to the center-of-mass motion.

More specifically, entanglement decoherence dynamics is fully determined by the spectral density of the environment, which is defined by 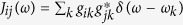. When the two continuous variables identically couple to the common environment: *g*_*ik*_ = *g*_*k*_, we have *J*_*ii*_(*ω*) = |*J*_12_(*ω*)|. In the center-of-mass frame, the spectral density is simply reduced to
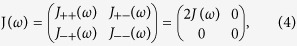
it shows that *J*_−−_(*ω*) = 0. This indicates that the environment synchronizes quantum as well as thermal fluctuations at the two identical parties, so that the relative motion experiences no fluctuation. Consequently, the corresponding entanglement contribution remains unchanged as the initial value for the relative motion (i.e. decoherence-free). The decoherence dynamics of the center-of-motion part is then fully controlled by the spectral component *J*_++_(*ω*) = 2*J*(*ω*). In the following, we consider the Ohmic-type spectral density^14^ which can simulate a large class of thermal bath,
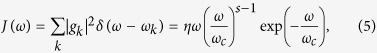
where *η* is a dimensionless coupling strength between the system and the environment, and *ω*_*c*_ is the cutoff of the environment spectrum. The parameter *s* classifies the environment as sub-Ohmic (*s* < 1), Ohmic (*s* = 1), or super-Ohmic (*s* > 1). Although we will focus on such a general environmental structure in this paper, the results are also valid to other spectral densities (other environmental structures) that do not be described by Eq. (5). 

The real-time dynamics of the initial state (2) can be solved directly from the reduced density matrix: 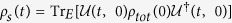, where 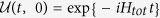 is the unitary evolution operator of the total system (the two entangled fields coupled their environment). The general solution is given by
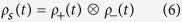
where *ρ*_+_(*t*) is the reduced density matrix of the center-of-mass motion. The relative motion remains a pure state *ρ*_−_(*t*) = |*ψ*_−_(*t*)〉〈*ψ*_−_(*t*)|. The explicit analytical solution can be found with the results
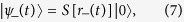

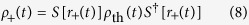
where 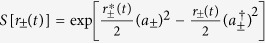 is the time-evolving squeezing operators of the center-of-mass motion and the relative motion of the two continuous variables, and Eq. (8) is a time-evolving squeezed thermal-like state^20^. Explicitly, the squeezing parameters
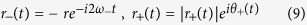
with 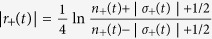 and 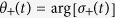. The nonequilibrium thermal-like state
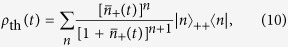
where 
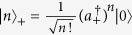
 is the Fock state of the center-of-mass variable. The average particle number in this state, 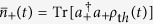, describes the nonequilibrium thermal fluctuations and satisfies the relation
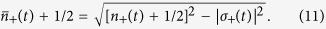


The factor 1/2 in the above equation is related to the zero-point energy fluctuation, and *n*_+_(*t*) and *σ*_+_(*t*) are respectively the photon intensity (the squeezed thermal fluctuation) and the two-photon correlation of the center-of-mass variable,
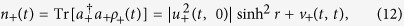

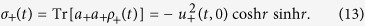


The functions *u*_+_(*t*, 0) and *v*_+_(*t*, *t*) are Schwinger-Keldysh’s retarded and correlated (fluctuation) Green functions in nonequilibrium many-body systems^21^,^22^,^23^ for the center-of-mass variable, and they obey the Kadanoff-Baym equations^23^ and the dissipation-fluctuation theorem^24^, respectively,
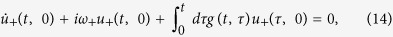

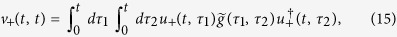
in which the integral kernels, 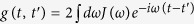 and 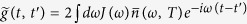, are fully determined by the spectral density of the environment. Here, 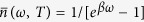 is the initial particle distributions in the environment.

The above analytical solutions show explicitly that the squeezed parameter in the relative-motion state *ρ*_−_(*t*) only takes a simple oscillation and is therefore decoherence-free. This is because the environment which equally couples to the two identical fields synchronizes both quantum (dissipation) and thermal fluctuations at the two identical parties so that the relative motion experiences no fluctuation, as a consequence of *J*_−−_(*ω*) = 0, see Eq. (4). On the other hand, all the environment-induced quantum (dissipation) and thermal fluctuations derive the center-of-mass motion part from an initial pure squeezed state into a squeezed thermal-like state *ρ*_+_(*t*), see Eq. (8) in which the squeezed operator *S*[*r*_+_(*t*)] acts on the nonequilibrium thermal-like state *ρ*_*th*_(*t*). The nonequilibrium thermal-like state *ρ*_*th*_(*t*), which is induced by the environment, is different from the usual equilibrium thermal state, because the averaged particle number 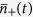 in *ρ*_*th*_(*t*) is not equal to the standard Bose-Einstein distribution 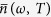 at the given frequency *ω* and the given equilibrium temperature *T*. Geometrically, *ρ*_*th*_(*t*) is also symmetrically distributed in terms of the quadrature components of the center-of-mass variable: 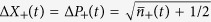, where 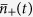 and 1/2 characterize the thermal and vacuum fluctuations, respectively. The squeezed parameter *r*_+_(*t*) is governed by both the quantum fluctuation *σ*_+_(*t*) and the thermal fluctuations embedded in the intensity *n*_+_(*t*) of the center-of-mass variable. The relation given by Eq. (11), 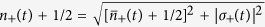 describes how the thermal-like state *ρ*_*th*_(*t*), is squeezed by the quantum fluctuation *σ*_+_(*t*) through the squeezing operator *S*[*r*_+_(*t*)]: 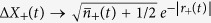 and 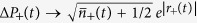. If *σ*_+_(*t*) → 0, we have *r*_+_(*t*) → 0 and *S*[*r*_+_(*t*)] → 1. Then the center-of-mass motion approaches to a thermal state governed by thermal fluctuations of the environment only.

### Nonequilibrium entanglement decoherence dynamics

The above analytical solutions show that the entanglement decoherence dynamics can be fully determined from the solution of Eq. (14) which provides the general quantum dissipation dynamics in open quantum systems^24^,
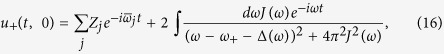
where the first term is contributed by the localized modes in the Fano-Anderson model^24^,^25^, with the frequencies 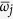 which are determined by the zeros of the function *z*(*ω*) ≡ *ω*_+_ − *ω* + Δ(*ω*), and the amplitude 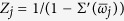 is the corresponding pole residue. Here 
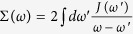
 is the self-energy, and 
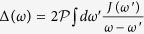
 denotes its principal value. This localized mode contributes a dissipationless dynamics. The second term in Eq. (16) always decays, which leads to the quantum dissipation (damping) dynamics in open systems. For the spectral density of Eq. (5), the localized mode occurs only when the dimensionless system-environment coupling strength is greater than the critical coupling strength *η*_*c*_(*s*) for a given environment characterized by *s*,
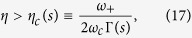
where 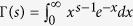 is the gamma function. On the other hand, the environment-induced fluctuations is characterized by *v*_+_(*t*, *t*), which is determined by Eq. (15) as a result of the generalized nonequilibrium fluctuation-dissipation theorem in the time-domain.

Now we can see that the center-of-mass state *ρ*_+_(*t*) contains various decoherence dynamics. For a given spectral density (fixed *s*), the state *ρ*_+_(*t*) in the strong-coupling regime (*η* > *η*_*c*_(*s*)) undergoes a partial decoherence process and then approach to a nonequilibrium state, due to the existence of the localized mode^25^,^26^,^27^,^28^,^29^,^30^. This property becomes obvious by looking at the initial state dependence, the squeezed parameter *r*-dependence in the time-dependent coefficients in Eqs (12, 13). This initial-state dependence is a manifestation of the strong non-Markovian memory effect, induced by the localized mode in the strong coupling, see Eq. (16). Thus, when *η* > *η*_*c*_(*s*), *ρ*_+_(*t*) becomes a nonequilibrium entanglement state that always depends on the initial state, even in the steady-state limit *t* → ∞. Only in the weak-coupling regime (*η* < *η*_*c*_(*s*)), the localized mode cannot be formed, and the Green function *u*_+_(*t*, 0) → 0 as *t* → ∞ so that *σ*_+_(*t*) → 0. Then the time-dependent squeezing parameter *r*_+_(*t*) → 0. As a result, the state *ρ*_+_(*t*) will approach to the thermal equilibrium state,
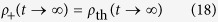
with the average particle number (thermal fluctuation) 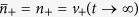. This state is independent of the initial squeezed state, and also contains no any entanglement. In other words, when *η* < *η*_*c*_(*s*), the system reaches equilibrium with its environment, and the entanglement of the center-of-mass state *ρ*_+_(*t*) is completely decohered away.

To be explicit, we should quantitatively study the entanglement decoherence dynamics by quantifying the entanglement between the two continuous variables in the two-mode entangled state, using the logarithmic negativity^31^. The logarithmic negativity has been widely used in the literature, based on the Peres-Horodecki positive partial transpose (PPT) criterion^32^ as a necessary and sufficient condition for the separability of bipartite Gaussian states. The entanglement degree of a bipartite Gaussian state is given by^31^:
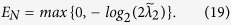
where 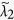 is the smaller one of the two symplectic eigenvalues of the covariant matrix. On the other hand, if the entangled state is a direct product of two entangled states, the total entanglement is also the sum of the logarithmic negativities from each state^31^. Meanwhile, we also find that for the case of the direct product state, like Eq. (6), in which one state undergoes a decoherence process and the other state is decoherence-free, one must calculate the entanglement from each state separately in the product and then add them together to get the correct total entanglement. If one uses the logarithmic negativity to calculate the entanglement directly from the original state, then the decoherence entanglement dynamics in the decoherence state will artificially decohere away some entanglement in the decoherence-free state, which is physically not possible. Thus, the total entanglement between the two original continuous variables in *ρ*_*s*_(*t*) is given by
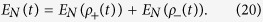
It is not difficult to find that for the initial state (2), the entanglement *E*_*N*_(*ρ*_+_(0)) = *E*_*N*_(*ρ*_−_(0)) = *r*/*ln*2, namely, the initial total entanglement is equally distributed between the relative-motion state |*ψ*_−_(0)〉 and the center-of-mass state |*ψ*_+_(0)〉.

The real-time dynamics of the entanglement for each part in Eq. (20) can be solved analytically. For the relative-motion state *ρ*_−_(*t*), because of its decoupling from the thermal reservoir, its entanglement remains *unchanged*, *E*_*N*_(*ρ*_−_(*t*)) = *r*/*ln*2. On the other hand, for the center-of-mass motion state *ρ*_+_(*t*), the time evolution of the entanglement can be determined through Eq. (19). It is not difficult to find the smaller symplectic eigenvalue 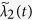 of the covariant matrix with respect to the two original continuous variables for *ρ*_+_(*t*),
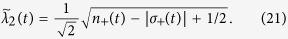


Then *E*_*N*_(*ρ*_+_(*t*)) can be computed from the nonequilibrium retarded and correlated Green functions *u*_+_(*t*, 0) and *v*_+_(*t*, *t*) through Eqs (12) and (13). The retarded Green function *u*_+_(*t*, 0) describes the dissipation dynamics, which is independent of the initial temperature of the environment, and manifests pure quantum correlations between the system and the environment^24^. The correlation Green function *v*_+_(*t*, *t*), the generalized nonequilibrium fluctuation-dissipation theorem, depends explicitly on the initial environment temperature, it describes the thermal fluctuations and the thermal-fluctuation-induced quantum fluctuations. The dynamics of the total entanglement of the two continuous variables with the initial state (2) is given by *E*_*N*_(*t*) = *E*_*N*_(*ρ*_+_(*t*)) + *r*/*ln*2.

We first consider the weak-coupling regime *η* < *η*_*c*_(*s*) at zero temperature, the function *u*_+_(*t*, *t*_0_) will decay to zero for *t* → ∞. Meanwhile, the correlation function *v*_+_(*t*, *t*) = 0 because 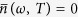 at zero temperature. Thus the time-dependent functions in Eqs (12, 13) lead to *σ*_+_(*t*) = 0 and *n*_+_(*t*) = 0 as *t* → ∞. As a result, Eq. (21) is reduced to 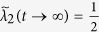 so that *E*_*N*_(*ρ*_+_(*t* → ∞)) = 0. Then the steady-state total entanglement *E*_*N*_(*t* → ∞) = *E*_*N*_(*ρ*_−_(*t*)) = *r*/*ln*2. This reproduces the result at zero temperature in the weak-coupling regime we obtained previously^33^. If the environment is initially at a finite temperature, because *u*_+_(*t* → ∞, 0) → 0 is always true for *η* < *η*_*c*_(*s*), we have *n*_+_(*t*) → *v*_+_(*t*, *t*) and *σ*_+_(*t*) → 0 so that
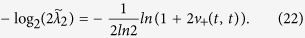


Also because *v*_+_(*t*, *t*) > 0 for any finite temperature, Eq. (22) gives always a negative value. According to the criterion (19), again the entanglement of *ρ*_+_(*t* → ∞) must go to zero. Indeed, Eq. (22) shows that thermal fluctuations speed up the entanglement decoherence. The steady-state entanglement *E*_*N*_(*ρ*_+_(*t* → ∞)) remains zero in the weak-coupling regime (*η* < *η*_*c*_(*s*)) for any initial environment temperature, and the steady-state total entanglement always equals to *E*_*N*_(*ρ*_−_(*t*)) = *r*/*ln*2.

Actually, the entanglement dynamics of Eq. (2) in the weak-coupling regime at finite temperature was previously studied in ref. 34 where it shows that the decoherence lets the total entanglement be less than one half of the initial total entanglement due to the thermal effect, see explicitly Fig. 8(b) in ref. 34. This is obviously a wrong result because the relative motion state *ρ*_−_(*t*) decouples from the environment such that the entanglement contained in *ρ*_−_(*t*) is decoherence-free. Thus, the steady-state total entanglement can never be less than *r*/*ln*2, as we shown above. The mistake made in ref. 34 comes from an improper calculation of the entanglement when the entangled state is a direct product of two states. The improper calculation in ref. 34 lets the entanglement decoherence dynamics of the center-of-mass motion artificially decohere away the entanglement in the relative motion state, while the later is however decoherence-free. This leads to the unphysical result of the steady-state total entanglement being less than *r*/*ln*2, as given in ref. 34.

On the other hand, in the strong-coupling regime (*η* > *η*_*c*_(*s*)), the propagating function *u*_+_(*t*, *t*_0_) will not decay to zero in the steady-state limit, due to the existence of localized states^24^,^25^. Thus the state *ρ*_+_(*t*) Eq. (8) cannot approach to a thermal equilibrium state because it maintains the initial-state dependence at *t* → ∞, see Eqs (12 and 13). Correspondingly, the entanglement dynamics undergoes a quantum phase transition as the system-environment coupling varying from the weak-coupling regime to the strong-coupling regime.

### Nonequilibrium quantum phase transition

**Figure 1:**
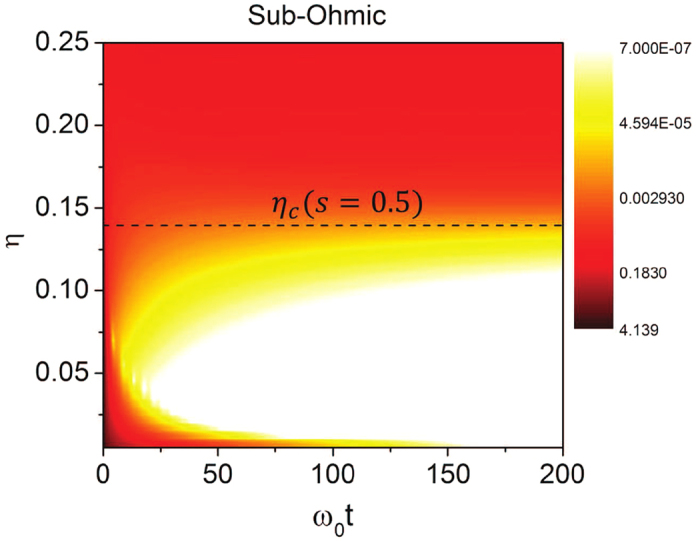
Real-time entanglement dynamics. The contour plot of the entanglement *E*_*N*_(*ρ*_+_(*t*)) (in log scale) as a function of the time and the system-environment coupling strength *η* at zero initial environment temperature for sub-Ohmic spectral density. The other parameters are taken as *ω*_*c*_ = 3*ω*_0_, *κ* = 0.5*ω*_0_ and *r* = 3.

Now, we shall numerically analyze the entanglement dynamics for different spectral densities to demonstrate the quantum phase transition discussed above. Taking the sub-Ohmic reservoir (*s* = 0.5) as an example, we present the real-time dynamics of the entanglement in Fig. 1. As it shows, in the weak-coupling regime *η* < *η*_*c*_(*s*) = 0.141 for *s* = 0.5, the entanglement of the center-of-mass motion is gradually decohered away. This is indeed true for all the three different Ohmic-type spectra given by Eq. (5). However, in the strong-coupling regime *η* > *η*_*c*_(*s*), the entanglement can be partially preserved, even in the long-time limit, due to the existence of the localized state which induces the long-time non-Markovian memory effect. This provides the real-time dynamics of the nonequilibrium quantum phase transition through entanglement decoherence as the system-environment coupling strength varies.

**Figure 2:**
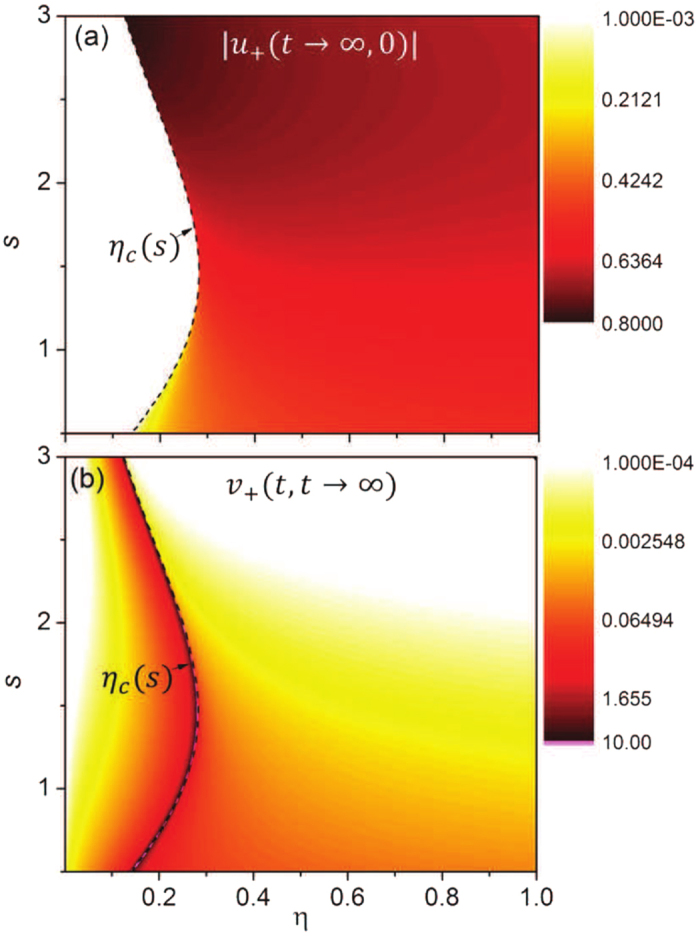
Localization and fluctuation dynamics. (a) The localization given by the retarded Green function |*u*_+_(*t* → ∞, 0)|, and (b) the fluctuation in terms of the correlated Green function *v*_+_(*t*, *t* → ∞) (in log scale) as a function of the coupling strength *η* and the spectral parameter *s*. The other parameters are taken as the same as in Fig. 1 but the initial environment temperature *T* = 0.1*ω*_0_.

To understand the origin of the quantum phase transition, we present the quantum dissipation and fluctuations, described by the steady-state retarded Green function *u*_+_(*t* → ∞, 0) and the correlation Green function *v*_+_(*t*, *t* → ∞), in Fig. 2. It shows that in the weak-coupling regime (*η* < *η*_*c*_(*s*)), the amplitude of the retarded Green function |*u*_+_(*t* → ∞, 0)| decays to zero, see the empty area in Fig. 2(a). In the strong-coupling regime (*η* > *η*_*c*_(*s*)), the localized mode occurs, then the amplitude of *u*_+_(*t* → ∞, 0) maintains a nonzero value, given by the color area in Fig. 2(a). This demonstrates clearly a quantum phase transition from dissipation dynamics to localization dynamics when the system-environment coupling passes through the critical coupling *η*_*c*_(*s*) for various spectral densities (different *s*). This phase transition is the first-order phase transition and is purely induced by quantum fluctuations. On the other hand, the corresponding steady-state fluctuation Green function *v*_+_(*t*, *t* → ∞) presented in Fig. 2(b) shows a huge amount of thermal fluctuations near the quantum critical transition line *η*_*c*_(*s*) (the dash line). When the coupling strength goes away from the critical regime around *η*_*c*_(*s*), the fluctuations decrease rapidly. This manifests the quantum criticality as a result of the competition between quantum fluctuations and thermal fluctuations.

**Figure 3:**
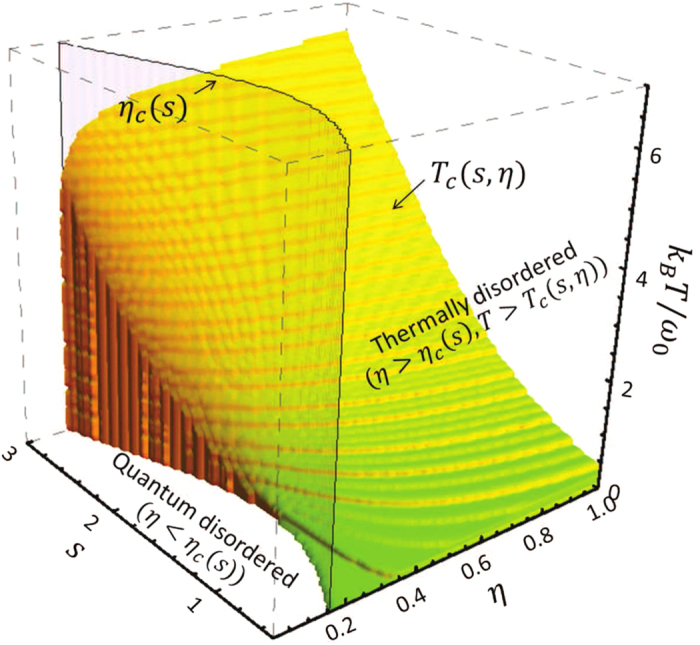
The 3-D phase diagram. A contour plot of entanglement degree *E*_*N*_(*ρ*_+_(*t* → ∞)) in 3-D space of the coupling strength *η*, the spectral parameter *s* and the initial reservoir temperature *T*. The other parameters are taken as the same as in Fig. 1.

With the above quantum criticality extracted from the fluctuation Green function *v*_+_(*t*, *t*), an entanglement phase diagram, in terms of the entanglement *E*_*N*_(*ρ*_+_(*t* → ∞)) as a function of the system-environment coupling *η*, the spectral parameter *s* and the initial environment temperature *T*, is presented in Fig. 3. As we see, at zero temperature, the *η* − *s* plane is separated into the quantum disordered (*η* < *η*_*c*_(*s*)) and quantum ordered (*η* > *η*_*c*_(*s*)) phases. When the initial environment temperature is nonzero, the competition between quantum fluctuations and thermal fluctuations shows up. As a result, the entanglement protected by the localization in the strong-coupling regime can be gradually decohered away by thermal fluctuations, and the thermal disordered phase with *E*_*N*_(*ρ*_+_(*t* → ∞)) = 0 is formed for *η* > *η*_*c*_(*s*) and *T* > *T*_*c*_(*s*, *η*), where *T*_*c*_(*s*, *η*) is a critical surface separating the quantum ordered phase and the thermal disordered phase, as shown in Fig. 3. Owing to the strong thermal fluctuation and small localized mode amplitude for the small value *s*, the thermal disordered phase starts to show up from the sub-Ohmic reservoir. Increasing the initial environment temperature will enhance thermal fluctuations such that the domain of the thermal disordered phase is enlarged, extending to the Ohmic and then super-Ohmic reservoir. It also shows that the transition from the quantum entangled phase to the thermal disordered phase is a continuous phase transition.

## Discussions

In this work, we find that entanglement decoherence, due to the environment-induced dissipation, localization and fluctuation dynamics, forms three different types of phases as the system-environment coupling strength *η*, the spectral parameter *s* and the initial environment temperature *T* vary: the dissipation-induced disentangled phase (phase I) in the weak-coupling regime *η* < *η*_*c*_(*s*) for arbitrary initial environment temperature; the quantum entangled phase (phase II, the colored part in Fig. 3) protected by the localized state in the strong-coupling regime *η* > *η*_*c*_(*s*) with *T* < *T*_*c*_(*s*, *η*), and the thermal disordered phase (phase III) as the result of thermal fluctuations dominating in the regime *η* > *η*_*c*_(*s*) and *T* > *T*_*c*_(*s*, *η*). The transition from phase I to phase II is the first-order quantum phase transition (corresponding to the transition of dissipation dynamics to localization dynamics^28^,^29^), while transition from phase II to phase III is a continuous phase transition. Also the results presented in this article can be applied to other open systems^24^. This provides a general procedure to explore nonequilibrium quantum phase transition through the experimental measurement of real-time entanglement decoherence dynamics in many-body systems.

## Additional Information

**How to cite this article**: Lin, Y.-C. *et al*. Non-equilibrium quantum phase transition via entanglement decoherence dynamics. *Sci. Rep.* **6**, 34804; doi: 10.1038/srep34804 (2016).
